# Are smokers who are regularly exposed to e-cigarette use by others more or less motivated to stop or to make a quit attempt? A cross-sectional and longitudinal survey

**DOI:** 10.1186/s12916-018-1195-3

**Published:** 2018-11-14

**Authors:** Sarah E. Jackson, Emma Beard, Susan Michie, Lion Shahab, Tobias Raupach, Robert West, Jamie Brown

**Affiliations:** 1 Department of Behavioural Science and Health, University College London, 1-19 Torrington Place, London, UK; 2 Department of Clinical, Educational and Health Psychology, University College London, London, UK; 3 Department for Cardiology and Pneumology, Göttingen University Medical Centre, Göttingen, Germany

**Keywords:** E-cigarettes, Motivation, Quit attempts, Prospective cohort study

## Abstract

**Background:**

Concerns have been raised that observing other people using e-cigarettes may undermine motivation to quit by renormalising smoking. This study aimed to explore associations between regular exposure to other people’s e-cigarette use and motivation to stop smoking and quit attempts in smokers.

**Methods:**

Data were from 12,787 smokers in England who participated in the Smoking Toolkit Study between November 2014 and May 2018. At baseline, respondents were asked whether anyone other than themselves regularly used an e-cigarette in their presence, whether they had made a quit attempt in the past year and how motivated they were to stop. Data at 6-month follow-up were available for 1580 respondents, who reported on whether they had attempted to quit in the past 6 months.

**Results:**

Smokers who reported regular exposure to e-cigarette use by others were more likely than those who did not to have tried to stop smoking in the past year (32.3% vs. 26.8%; unadjusted RR 1.21, 95% CI 1.11–1.31) and have high motivation to quit (16.6% vs. 14.2%; unadjusted RR 1.17, 95% CI 1.05–1.30) but were not significantly more or less likely to make a quit attempt over the subsequent 6 months (34.4% vs. 31.3%; unadjusted RR 1.10, 95% CI 0.88–1.38). In models that adjusted for participants’ own current e-cigarette use and unadjusted and adjusted models excluding current e-cigarette users from the sample, there were no significant associations between exposure to e-cigarette use by others and past quit attempts (RR 0.97–1.00), high current motivation to quit (RR 0.97–1.00) or prospective quit attempts (RR 0.94–1.12). In contrast, exposure to use of cigarettes was associated with low motivation to quit even after adjustment (RR 0.89) but not with quit attempts. Participants’ own use of e-cigarette was strongly associated with high motivation to quit (RR 1.95) and past quit attempts (RR 2.14) and appeared to account for the bivariate associations with reported exposure to e-cigarettes.

**Conclusion:**

Smokers who report regular exposure to other people using e-cigarettes are more likely to report past quit attempts and high current motivation to quit, but there does not appear to be an independent association with motivation or quit attempts after adjustment for their own current use of e-cigarettes. In contrast, reported exposure to other people using cigarettes was independently and negatively associated with high motivation.

**Electronic supplementary material:**

The online version of this article (10.1186/s12916-018-1195-3) contains supplementary material, which is available to authorized users.

## Introduction

E-cigarette use is prevalent in England, with approximately 20% of smokers currently using them and around 13% of smokers currently using at least daily [1]. Apart from their impact on the smoking behaviour of users, these devices may affect non-users in ways that are hard to predict but which may nevertheless be important for public health [2–7]. Studies have found that exposure to other tobacco smokers is a predictor of relapse [8–10] and theories of smoking addiction and cessation describe how cues play an important role in the maintenance and resumption of smoking [11]. There are concerns that exposure to e-cigarette use by others may be ‘renormalising’ smoking [12–16], which could reduce smokers’ motivation to try to stop. However, it is also possible that seeing other people use e-cigarettes may motivate smokers to try to use these devices to help them quit. Indeed, qualitative work has indicated that many people interpret use of e-cigarettes as indicating that the user is trying to reduce or stop smoking [17]. This study examined associations between exposure to e-cigarette use by others and motivation to quit in smokers.

Evidence on the impact of exposure to e-cigarette use by others is scant, but a few experimental studies suggest that it may have an adverse influence on smokers currently attempting to quit smoking. In two studies of young adult smokers, exposure to e-cigarette use by others increased desire and urges to use e-cigarettes and smoke combustible cigarettes [18, 19]. In another study, smokers maintained their gaze longer on pictures of e-cigarettes than on neutral pictures in a visual probe task, indicating that current smokers have an attentional bias for e-cigarette cues [20]. However, sample sizes were small and results cannot be presumed to generalise to the general population of smokers. In addition, behavioural outcomes were not assessed so the extent to which effects of e-cigarette exposure translate into differences in quitting behaviours is not known. To our knowledge, no studies have explored the impact of e-cigarette exposure in a real-world setting, and none have examined associations with motivation to stop smoking or quit attempts.

This study aimed to examine associations between reported regular exposure to e-cigarette use and (i) motivation to quit and (ii) attempts to stop smoking across smokers participating in a large population-based survey. Specifically, we were interested in:

1. The cross-sectional association between reported regular exposure to e-cigarette use and a quit attempt in the last 12 months.
2. The cross-sectional association between reported regular exposure to e-cigarette use and the prevalence of high current motivation to stop smoking.
3. The prospective association between reported regular exposure to e-cigarette use and the incidence of any quit attempt in the following 6 months.

## Method

### Design

The Smoking Toolkit Study is an ongoing monthly survey designed to provide information about smoking prevalence and behaviour and factors associated with cessation in England at a population level [21]. The study uses hybrid random location and quota sampling to select a new sample of approximately 1700 adults aged ≥ 16 years each month. Participants complete a face-to-face computer-assisted survey with a trained interviewer. All smokers participating between March 2014 and September 2016 who agreed to be re-contacted were telephoned 6 months after the baseline assessment to complete a follow-up survey. Data were pooled across waves to produce one sample for analysis. Full details of the baseline methods are available elsewhere, and comparisons with national data indicate that key variables such as socio-demographics and smoking prevalence are nationally representative [21].

### Study population

For the present study, we used data from respondents to the survey in the period from November 2014 (the first wave to ask about regular exposure to e-cigarette users) to May 2018 (the latest wave for which data were available). Participants were included in the analyses if they reported smoking cigarettes (manufactured or hand-rolled) or any other tobacco product (e.g. pipe, cigar) daily or occasionally at the time of the baseline survey and had complete data on all variables of interest. Cross-sectional analyses were carried out on the full available sample (*n* = 12,787), and prospective analyses were carried out on the subsample for whom follow-up data were available (*n* = 1580).

### Measures

#### Measurement of e-cigarette exposure

The independent variable of interest was reported regular exposure to e-cigarette use, measured at baseline. Participants were asked at baseline ‘Other than yourself, does anyone regularly smoke cigarettes or use an e-cigarette in your presence, such as at your home, work, car or other places that you visit regularly?’. Responses were coded 0 for those who responded ‘no—neither cigarettes nor e-cigarettes’ or ‘yes—cigarettes only’ and 1 for those who responded ‘yes—e-cigarettes only’ or ‘yes—both cigarettes and e-cigarettes’.

#### Measurement of outcomes

The main outcome measures were quit attempts made in the past 12 months measured at baseline (‘past quit attempts’), current motivation to stop smoking measured at baseline (‘motivation to stop’) and prospective quit attempts at 6-month follow-up (‘prospective quit attempts’).

Past quit attempts were assessed at baseline with the question ‘How many serious attempts to stop smoking have you made in the past 12 months? By serious I mean you decided that you would try to make sure you never smoked again.’ This item was coded 0 for smokers who responded that they had not made a quit attempt and 1 for those who reported one or more quit attempts.

Motivation to stop smoking was assessed at baseline using the Motivation To Stop Scale [22], a single-item measure with seven response options representing increasing motivation to quit: (1) ‘I don’t want to stop smoking’, (2) ‘I think I should stop smoking but don’t really want to’, (3) ‘I want to stop smoking but haven’t thought about when’, (4) ‘I REALLY want to stop smoking but I don’t know when I will’, (5) ‘I want to stop smoking and hope to soon’, (6) ‘I REALLY want to stop smoking and intend to in the next 3 months’ and (7) ‘I REALLY want to stop smoking and intend to in the next month’. To aid interpretation, responses were dichotomised to reflect high (6–7) vs. low (1–5) motivation to stop smoking [23].

In the subsample who were invited and responded to the 6-month follow-up survey, prospective quit attempts were assessed at 6-month follow-up with the question ‘How many serious attempts to stop smoking have you made in the last 6 months? By serious I mean you decided that you would try to make sure you never smoked again.’ Responses were coded 0 for those who had not made a quit attempt and 1 for those who reported at least one quit attempt in the last 6 months (i.e. since the baseline survey).

#### Measurement of potential confounders

All potential confounders were selected a priori. Participants provided data at baseline on age, sex and social grade (ABC1, which includes managerial, professional and intermediate occupations, vs. C2DE, which includes small employers and own-account workers, lower supervisory and technical occupations and semi-routine and routine occupations, never workers and long-term unemployed). We included survey year to take account of changes in the availability and regulation of e-cigarettes over the study period. Regular exposure to cigarette smoking by others was measured using the item assessing exposure to e-cigarette use described above in order to evaluate the impact of e-cigarette exposure over and above exposure to cigarette smoking. We also adjusted for participants’ own current use of e-cigarettes, assessed with the item ‘Are you using any of the following either to help you stop smoking, to help you cut down or for any other reason at all?’, with those responding ‘yes’ to the option ‘electronic cigarette’ coded 1 and those responding ‘no’ coded 0.

### Statistical analyses

The analysis plan was registered on the Open Science Framework before data analysis (http://osf.io/45mux/).

We examined potential differences in baseline characteristics between the baseline sample and the subset of participants who completed the 6-month follow-up using *t* tests for continuous variables and chi-square tests for categorical variables.

We used log-binomial regression to analyse the dichotomous cross-sectional and prospective outcomes and calculate the associated relative risk (RR) and 95% confidence intervals (CIs). In the baseline sample of current smokers, we tested associations between e-cigarette exposure and past quit attempts and high current motivation to stop smoking. In the subset of participants who reported current smoking at baseline and responded to the 6-month follow-up questionnaire, we tested the association between e-cigarette exposure and prospective quit attempts. For all outcomes, we report bivariate associations with e-cigarette exposure and independent associations adjusting for age, sex, social grade, reported regular exposure to cigarette smoking by others, own e-cigarette use and survey year.

In order to evaluate the extent to which including current e-cigarette users in our sample affected the results, we examined associations with participants’ own e-cigarette use in adjusted models and performed a sensitivity analysis excluding e-cigarette users from the sample. Where non-significant associations were found, we calculated Bayes factors (BF; planned a priori) to examine whether these associations can best be characterised as evidence of no effect, evidence of an effect or whether data were insensitive to detect an effect [24, 25]. Alternative hypotheses were represented by half-normal distributions, and the expected effect size was set to RR = 0.7 based on previous research into other important barriers to quit attempts [26]. We also calculated BFs with the expected effect size set to RR = 0.9 to test sensitivity to detect small negative effects of exposure to e-cigarette use by others on motivation and quit attempts. To test sensitivity of the data to detect positive effects, we calculated BFs using the equivalent effect sizes in the opposite direction (RR = 1.3 and RR = 1.1). In order to calculate BFs using the online calculator specified in our protocol, which only allows expected effects modelled by a half-normal distribution to be expressed positively, we reran each model reversing the categorisation of e-cigarette exposure (0 = exposed, 1 = not exposed) and entered expected effect sizes as the natural log of 1.3 and 1.1 (as opposed to 0.7 and 0.9), respectively. A BF ≥ 3 can be interpreted as substantial evidence for the alternative hypothesis (and against the null), while a BF of ≤ 1/3 can be interpreted as evidence for the null hypothesis. BFs between 1/3 and 3 suggest that the data are insensitive to distinguish the alternative hypothesis from the null [24, 27].

In addition to our pre-registered analyses, we performed unplanned sensitivity analyses testing associations between regular exposure to e-cigarettes by others and past quit attempts, high current motivation to quit and prospective quit attempts using a four-category exposure variable, coded in line with the way the question was asked in the survey: (i) cigarettes only, (ii) e-cigarettes only, (iii) both cigarettes and e-cigarettes and (iv) neither cigarettes nor e-cigarettes (reference category). We report bivariate associations and independent associations adjusting for age, sex, social grade, own e-cigarette use and survey year.

All analyses were performed using SPSS version 25 with the exception of the Bayes factors which were calculated using an online calculator (http://www.lifesci.sussex.ac.uk/home/Zoltan_Dienes/inference/Bayes.htm).

## Results

A total of 13,209 smokers were surveyed between November 2014 and May 2018, of whom 12,787 (96.8%) provided complete data on all baseline variables. Sample characteristics are summarised in Table 1. A subset of 1580 participants (21.9% of those participating in eligible waves) completed the 6-month follow-up survey. Comparison of those who did and did not respond to the 6-month follow-up indicated that responders tended to be older and from a higher social grade than non-responders, and fewer were exposed to cigarette smoking by others. There were no significant differences by sex, exposure to e-cigarette use by others, own use of e-cigarettes, past quit attempts or high motivation to stop smoking at baseline.

**Table 1:** Sample characteristics

	Baseline sample (*n* = 12,787)	Follow-up sample (*n* = 1580)	p*
Demographic characteristics			
Age, % (*n*)			
16–24	19.1 (2439)	11.9 (188)	< 0.001
25–34	19.5 (2495)	13.1 (207)	–
35–44	15.9 (2033)	14.0 (221)	–
45–54	16.9 (2163)	20.5 (324)	–
55–64	14.7 (1883)	20.6 (325)	–
65+	13.9 (1774)	19.9 (315)	–
Sex, % (*n*)			
Men	53.2 (6799)	52.5 (829)	0.227
Women	49.8 (5988)	47.5 (751)	–
Social grade, % (*n*)			
ABC1	40.3 (5156)	43.5 (687)	< 0.001
C2DE	59.7 (7631)	56.5 (893)	–
Smoking characteristics			
Exposure to e-cigarette use by others, % (*n*)			
No	74.2 (9484)	74.6 (1179)	0.688
Yes	25.8 (3303)	25.4 (401)	–
Exposure to cigarette smoking by others, % (*n*)			
No	33.6 (4294)	36.0 (569)	0.002
Yes	66.4 (8493)	64.0 (1011)	–
Own e-cigarette use^†^, % (*n*)			
No	80.4 (10284)	79.0 (1248)	0.416
Yes	19.6 (2503)	21.0 (332)	–
Past quit attempt, % (*n*)			
No	71.8 (9178)	71.0 (1122)	0.203
Yes	28.2 (3609)	29.0 (458)	–
High motivation to stop smoking, % (*n*)			
No	85.1 (10887)	85.0 (1343)	0.605
Yes	14.9 (1900)	15.0 (237)	–
Prospective quit attempt, % (*n*)			
No	NA	67.9 (1073)	–
Yes	NA	32.1 (507)	–

*Comparison of participants who did and did not provide follow-up data

^†^Assessed with the question: ‘Are you using any of the following either to help you stop smoking, to help you cut down or for any other reason at all?’

Around a quarter (25.8%) of participants reported regular exposure to e-cigarette use by others. Smokers who were regularly exposed to e-cigarette use by others were more likely to have made a quit attempt in the past year (32.3%) than those who were not (26.8%; RR 1.21, 95% CI 1.11–1.31, *p* < 0.001). Those who were regularly exposed to e-cigarette use by others were also more likely to report high motivation to stop smoking at baseline (16.6% vs. 14.2%; RR 1.17, 95% CI 1.05–1.30, *p* = 0.005). However, exposure to e-cigarette use by others was not significantly associated with making a quit attempt 6 months later among participants who responded to follow-up (34.4% vs. 31.3%; RR 1.10, 95% CI 0.88–1.38, *p* = 0.410).

After adjustment for age, sex, social grade, exposure to cigarette smoking by others, own use of e-cigarettes and survey year (Table 2), there was no significant association between exposure to e-cigarette use by others and past quit attempts or high motivation to stop smoking. The association between exposure to e-cigarette use by others and prospective quit attempts remained non-significant. Participants’ own use of e-cigarettes was the variable most strongly associated with past quit attempts, high motivation to stop smoking and prospective quit attempts. In addition, younger and female smokers were more likely to have made a quit attempt in the past year and to report high motivation to stop smoking. Those from a lower social grade and those who reported regular exposure to cigarette smoking by others were less likely to be highly motivated to stop smoking. Older smokers were less likely to make a quit attempt over the following 6 months.

**Table 2:** Associations between exposure to e-cigarette use in others and past quit attempts, high motivation to stop smoking and prospective quit attempts

	Past quit attempts (*n* = 12,787)			High motivation to stop smoking (*n* = 12,787)			Prospective quit attempts (*n* = 1580)		
	% [95% CI]	RR_adj_ [95% CI]	*p*	% [95% CI]	RR_adj_ [95% CI]	*p*	% [95% CI]	RR_adj_ [95% CI]	*p*
Exposure to e-cigarette use by others									
No	26.8 [25.9–27.7]	1.00	–	14.2 [13.6–15.0]	1.00	–	31.3 [28.7–34.0]	1.00	–
Yes	32.3 [30.8–33.9]	0.97 [0.89–1.06]	0.546	16.6 [15.4–17.9]	0.99 [0.88–1.11]	0.839	34.4 [29.9–39.2]	0.94 [0.74–1.20]	0.627
Age									
16–24	29.8 [28.0–31.6]	1.00	–	13.6 [12.3–15.0]	1.00	–	38.3 [31.7–45.4]	1.00	–
25–34	33.1 [31.3–35.0]	1.10 [0.98–1.24]	0.094	17.1 [15.6–18.6]	1.24 [1.06–1.45]	0.007	40.1 [33.7–46.9]	1.05 [0.72–1.53]	0.783
35–44	32.0 [30.0–34.0]	1.05 [0.93–1.19]	0.430	18.6 [17.0–20.4]	1.34 [1.14–1.57]	< 0.001	35.7 [29.7–42.3]	0.91 [0.62–1.33]	0.626
45–54	27.0 [25.1–28.9]	0.89 [0.79–1.01]	0.067	16.4 [14.9–18.0]	1.17 [0.99–1.37]	0.066	35.2 [30.2–40.5]	0.92 [0.65–1.30]	0.629
55–64	24.9 [23.0–26.9]	0.83 [0.73–0.95]	0.006	13.2 [11.8–14.8]	0.96 [0.80–1.15]	0.652	25.5 [21.1–30.6]	0.67 [0.47–0.97]	0.035
65+	20.1 [18.3–22.0]	0.71 [0.62–0.82]	< 0.001	9.1 [7.8–10.5]	0.68 [0.56–0.83]	< 0.001	24.1 [19.7–29.2]	0.65 [0.45–0.96]	0.028
Sex									
Men	26.9 [25.8–27.9]	1.00	–	13.7 [12.9–14.6]	1.00	–	31.7 [28.6–35.0]	1.00	–
Women	29.8 [28.6–31.0]	1.10 [1.02–1.19]	0.012	16.2 [15.3–17.1]	1.16 [1.05–1.28]	0.003	32.5 [29.2–35.9]	1.02 [0.83–1.24]	0.877
Social grade									
ABC1	29.8 [28.5–31.0]	1.00	–	16.7 [15.8–17.8]	1.00	–	34.6 [31.2–38.3]	1.00	–
C2DE	27.2 [26.2–28.2]	0.93 [0.86–1.00]	0.053	13.6 [12.8–14.4]	0.83 [0.75–0.91]	< 0.001	30.1 [27.2–33.2]	0.87 [0.71–1.07]	0.175
Exposure to cigarette smoking by others									
No	27.8 [26.4–29.1]	1.00	–	15.7 [14.6–16.8]	1.00	–	29.7 [26.1–33.6]	1.00	–
Yes	28.5 [27.5–29.4]	0.99 [0.91–1.08]	0.777	14.4 [13.7–15.2]	0.89 [0.80–0.99]	0.033	33.4 [30.6–36.4]	1.05 [0.83–1.31]	0.703
Own e-cigarette use^†^									
No	23.0 [22.2–23.8]	1.00	–	12.4 [11.8–13.1]	1.00	–	29.2 [26.8–31.8]	1.00	–
Yes	49.8 [47.8–51.7]	2.14 [1.97–2.33]	< 0.001	25.0 [23.3–26.7]	1.95 [1.75–2.18]	< 0.001	42.8 [37.6–48.1]	1.44 [1.14–1.83]	0.002
Survey year	–	1.00 [0.97–1.04]	0.925	–	1.02 [0.97–1.07]	0.425	–	0.95 [0.76–1.19]	0.537

*RR* risk ratio, *CI* confidence interval

^†^Assessed with the question: ‘Are you using any of the following either to help you stop smoking, to help you cut down or for any other reason at all?’

Sensitivity analyses that did not include participants’ own use of e-cigarettes as a covariate in the adjusted models produced results that were similar to the unadjusted statistics, showing significant associations between exposure to e-cigarette use by others and past quit attempts (RR_adj_ 1.18, 95% CI 1.09–1.29, *p* < 0.001) and high motivation to stop smoking (RR_adj_ 1.17, 95% CI 1.05–1.31, *p* = 0.006) and no significant association for prospective quit attempts (RR_adj_ 1.02, 95% CI 0.80–1.29, *p* = 0.900).

Participants who were regularly exposed to e-cigarette use by others were more than twice as likely as those who were not to report using e-cigarettes themselves (34.8% vs. 14.3%, *χ*^2^(1) = 657.20, *p* < 0.001). When e-cigarette users (*n* = 2503) were excluded from the sample, there was no significant association between exposure to e-cigarette use by others and past quit attempts (23.0% in those exposed to e-cigarette use vs. 23.0% in those who were not) in unadjusted (RR 1.00, 95% CI 0.90–1.12, *p* = 0.953) or adjusted models (RR_adj_ 0.97, 95% CI 0.87–1.09, *p* = 0.603). Likewise, there was no significant association with high motivation to stop smoking (12.4% vs. 12.4%; RR 1.00, 95% CI 0.86–1.15, *p* = 0.952; RR_adj_ 0.97, 95% CI 0.84–1.13, *p* = 0.724) or prospective quit attempts (31.9% vs. 28.6%; RR 1.12, 95% CI 0.84–1.48 *p* = 0.446; RR_adj_ 1.03, 95% CI 0.77–1.39, *p* = 0.838).

Based on expected effect sizes of 0.7 and 1.3, the majority of Bayes factors indicated that the results from the adjusted models and sensitivity analyses relating to past quit attempts and high motivation to stop smoking provided moderate evidence for the null hypothesis (BF range 0.22–0.39 for an effect size of 0.7, 0.09–0.26 for an effect size of 1.3; Additional file 1: Table S1); that is, reported regular exposure to e-cigarette use by others was not associated with decreased or increased likelihood of making a quit attempt over the past year or high motivation to stop smoking at baseline. All data relating to prospective quit attempts also marginally favoured the null hypothesis but were insensitive to detect an effect in some analyses (BF range 0.20–0.65 for an effect size of 0.7, 0.29–0.91 for an effect size of 1.3). Repeating these calculations with BFs based on the expected effect sizes of 0.9 and 1.1, all outcomes were insensitive to detect small effects (range 0.39–1.18), with the exception of the adjusted model for past quit attempts in the full baseline sample which provided moderate evidence for the null hypothesis (BF 0.24).

Sensitivity analyses that used a four-category exposure variable that compared participants reporting no regular exposure to either cigarettes or e-cigarettes with those reporting regular exposure to (i) cigarettes only, (ii) e-cigarettes only and (iii) both cigarettes and e-cigarettes revealed no notable differences in results. In unadjusted models, relative to those with no exposure, those regularly exposed only to e-cigarette use by others were significantly more likely to report a past quit attempt (RR 1.33, 95% CI 1.07–1.63) and high current motivation to quit (RR 1.45, 95% CI 1.13–1.88), but there was no significant association with prospective quit attempts (RR 0.80, 95% CI 0.42–1.49). After adjustment for covariates, there was no significant association between regular exposure to e-cigarette use by others and past quit attempts (RR_adj_ 1.05, 95% CI 0.85–1.30), high current motivation to quit (RR_adj_ 1.19, 95% CI 0.92–1.54) or prospective quit attempts (RR_adj_ 0.68, 95% CI 0.36–1.28). Complete results for the unadjusted and adjusted models are available in Additional file 2: Table S2.

## Discussion

These analyses show that smokers who are regularly exposed to other people using e-cigarettes were more motivated to stop smoking and were more likely to have tried to quit, but this was not independent of their own use of e-cigarettes; in fact, there was moderate evidence for no independent association between exposure to e-cigarette use by others and motivation to stop smoking or quit attempts.

This is the first study, to our knowledge, that has assessed the impact of exposure to other people’s e-cigarette use on smokers’ motivation and behaviour. One in four smokers reported that someone else regularly used e-cigarettes in their presence. In contrast to experimental studies that have reported increased desire and urges to smoke in smokers exposed to e-cigarette use by others [18, 19], our unadjusted models were suggestive of beneficial effects of e-cigarette exposure. Smokers who were regularly exposed to e-cigarette use by others were around 20% more likely than those not regularly exposed to report being highly motivated to stop smoking in the next 3 months or to have made a serious quit attempt in the last year. One possible explanation for these discrepant findings is that the outcomes of interest in the experimental studies (desire, urges) are momentary and vary in response to features of the current environment, whereas outcomes in our study were likely to be more stable. It could also be that the impact of single exposure to an unknown person using an e-cigarette differs to the impact of regular exposure to e-cigarette use by a close social connection such as a friend, relative or colleague, particularly if the latter is known to have used e-cigarettes to successfully reduce or stop smoking [28–30]. Thus, while seeing someone use an e-cigarette may momentarily increase desire to smoke, repeated exposure to other people’s e-cigarette use may draw non-users’ attention to the potential utility of these devices in helping them to quit smoking, increasing their motivation to make a quit attempt.

When we adjusted for potential confounding by age, sex, social grade, survey year, reported regular exposure to cigarette smoking by others and participants’ own e-cigarette use, there was no association between exposure to e-cigarette use by others and motivation or quit attempts, although exposure to cigarette smoking by others was independently associated with reduced high motivation to quit. The differences we observed in the unadjusted models were driven primarily by participants’ own e-cigarette use, which was strongly associated with motivation and quit attempts and more commonly reported by smokers who were regularly exposed to e-cigarette use by others. Among smokers who did not use e-cigarettes at all, there was no evidence that exposure to other people using e-cigarettes affected motivation or quit attempts. It is possible that excluding e-cigarette users from these analyses might have overrepresented smokers who have little or no interest in e-cigarettes and who therefore might be less influenced by other people using e-cigarettes, thereby underestimating the association with e-cigarette exposure. The same pattern of results was seen when we retained e-cigarette users in the sample and adjusted for participants’ own use of e-cigarettes. These findings should ease concerns about potential adverse effects of e-cigarettes on non-users’ motivation and efforts to stop smoking [15, 16, 31].

While we found no evidence that exposure to other people using e-cigarettes adversely affected smokers’ motivation to stop or attempts to quit, there was a strong association between exposure to e-cigarette use by others and own e-cigarette use. Smokers who were regularly exposed to other people using e-cigarettes were more than twice as likely as those who were not to report using e-cigarettes themselves. While we cannot disentangle the direction of this association because it was not investigated longitudinally, previous research into network phenomena in smoking behaviour suggests that exposure to e-cigarette use by others might encourage non-users to take up using the devices. In a landmark paper that studied a densely interconnected social network of around 12,000 people, Christakis et al. demonstrated that smoking behaviour spreads through social connections, such that people are substantially more likely to smoke if they have a family member, friend or colleague who smokes and more likely to quit if a close social connection quits [32]. The effective international mass media campaign ‘Stoptober’ was predicated on this social contagion [33]. It is possible that the same might be true for e-cigarettes, such that having a close social connection who uses an e-cigarette may increase a person’s likelihood of using the devices themselves. In a large sample of US adults who reported ever trying e-cigarettes, friends or family members using or offering e-cigarettes was cited as one of the most common reasons for trying e-cigarettes [34], and knowing people who use e-cigarettes has been shown to predict e-cigarette use in adolescent samples [35, 36]. Qualitative studies of e-cigarette users have found that the majority were introduced to e-cigarettes by friends or family who already used them [37, 38].

This study had a number of strengths, including a large sample that was representative of smokers in the English population. We used data from monthly surveys spanning 3.5 years, which limited potential bias from seasonal differences in the rate of quit attempts. Models were adjusted for a range of potential confounders that are often either not assessed in other surveys or omitted from analyses. However, there were also limitations. The assessment of quit attempts relied on recall of the last 12 months for past quit attempts and the last 6 months for prospective quit attempts, introducing scope for bias. Due to differential drop-out, the follow-up sample was not representative of the baseline sample (typically being older and from a higher social grade), which may have biased the results. In addition, the findings may not generalise to populations outside of England where tobacco control policies and regulations (e.g. the legal status of e-cigarettes) differ. The wording of the item used to assess exposure to e-cigarette use by others did not define ‘regular’ so this measure relies on each respondent’s interpretation of regular exposure. Experimental research has indicated that smokers have an attentional bias for e-cigarette cues [20]. We did not adjust for tobacco dependence, and it is possible that highly dependent smokers might react more strongly to e-cigarette cues than smokers who are less dependent. However, previous research has found that while dependence is predictive of whether a quit attempt is successful, it is usually not associated with whether a quit attempt is made [26]. A priori we therefore decided there was not sufficient justification to warrant its inclusion as a potential confounder. Participants’ own e-cigarette use was assessed with an item that asked about use ‘to help you stop smoking, to help you cut down or for any other reason at all’; it is possible that the emphasis on quitting or reducing smoking may have accounted in part for the strong association we observed between own use of e-cigarettes and motivation to quit and quit attempts. However, estimates of current e-cigarette use in the Smoking Toolkit Study are extremely close to those produced by the Office for National Statistics using the Opinions and Lifestyle Survey, which uses a less prescriptive assessment [39]. Exposure to e-cigarette cues other than vaping itself (e.g. e-cigarette advertisements) was not assessed; this means that e-cigarette exposure may have been underestimated, and this study cannot inform any discussions about e-cigarette advertisements which might have an effect on smokers’ motivation to stop or make a quit attempt. E-cigarette advertising in England has been tightly regulated by the EU Tobacco Product Directive since 2016. Sensitivity analyses using Bayes factors indicated that the data favoured the null hypothesis but were insensitive to detect small effects, so it is possible that regular exposure to e-cigarettes has small effects on smokers’ motivation and attempts to stop smoking that were not detected in this study. It is also possible that there are effects beyond motivation and attempts. Further, qualitative research may be able to identify other important outcomes.

## Conclusions

In conclusion, the concern that the surge in popularity of e-cigarettes may be renormalising smoking in England and that this may discourage smokers from trying to stop appears unsupported by our findings. Our results indicate that, in fact, smokers who are regularly exposed to other people using e-cigarettes are more likely to be highly motivated to stop smoking and more likely to have made a recent quit attempt than smokers who do not regularly encounter people using e-cigarettes. A key factor underpinning these differences seems to be that smokers who are regularly exposed to e-cigarette use by others are more likely to use e-cigarettes themselves. Independent of smokers’ own use of e-cigarettes, regular exposure to other people using e-cigarettes appears to have little impact on how motivated smokers are to stop and whether they make a quit attempt. While it is important to consider these results in light of the study’s limitations, in particular relating to self-reported items, having found no evidence for an adverse impact of e-cigarette exposure on non-users, these findings should offer some reassurance in terms of the wider public health impact of e-cigarettes, particularly given evidence that the alternative, cigarette smoking, may reduce other smokers’ motivation to quit.

## Additional files

Additional file 1: **Table S1.** Bayes factors for non-significant results. (DOCX 17 kb)

Additional file 2: **Table S2.** Unadjusted and adjusted associations between exposure to e-cigarette use in others and past quit attempts, high motivation to stop smoking and prospective quit attempts. (DOCX 21 kb)

## Abbreviations

BF: Bayes factor
CI: Confidence interval
RR: Relative risk
