# Supplementary material for: Are smokers who are regularly exposed to e-cigarette use by others more or less motivated to stop or to make a quit attempt? A cross-sectional and longitudinal survey
Source: BMC Med. 2018 Nov 14;16:206. doi: 10.1186/s12916-018-1195-3 (PMC6234626; doi:10.1186/s12916-018-1195-3)
Supplement: Supplementary file 1 — Table S1. Bayes factors for non-significant results. (DOCX 17 kb) [file 12916_2018_1195_MOESM1_ESM.docx]

| **Table S1** Bayes factors for non-significant results | | | | | | |
| --- | --- | --- | --- | --- | --- | --- |
| **Outcome** | **Sample** | **Model adjustment** | **BF (effect size 0.7)** | **BF (effect size 0.9)** | **BF (effect size 1.1)** | **BF (effect size 1.3)** |
| Past quit attempts | Baseline | All covariates | 0.32* | 0.74 | 0.24* | 0.09** |
| Past quit attempts | Baseline excluding e-cigarette users | Unadjusted | 0.22* | 0.53 | 0.53 | 0.22* |
| Past quit attempts | Baseline excluding e-cigarette users | All covariates | 0.34 | 0.77 | 0.39 | 0.15* |
| High motivation to stop smoking | Baseline | All covariates | 0.25* | 0.60 | 0.48 | 0.19* |
| High motivation to stop smoking | Baseline excluding e-cigarette users | Unadjusted | 0.26* | 0.59 | 0.59 | 0.26* |
| High motivation to stop smoking | Baseline excluding e-cigarette users | All covariates | 0.39 | 0.82 | 0.45 | 0.19* |
| Prospective quit attempts | Follow-up | Unadjusted | 0.20* | 0.46 | 1.18 | 0.82 |
| Prospective quit attempts | Follow-up | All covariates | 0.65 | 1.01 | 0.61 | 0.29* |
| Prospective quit attempts | Follow-up | All covariates excluding own use of e-cigarettes | 0.39 | 0.75 | 0.87 | 0.49 |
| Prospective quit attempts | Follow-up excluding e-cigarette users | Unadjusted | 0.27* | 0.57 | 1.18 | 0.91 |
| Prospective quit attempts | Follow-up excluding e-cigarette users | All covariates | 0.43 | 0.77 | 0.93 | 0.59 |
| BF = Bayes factor.  Bayes factors between 1/30 and 1/10 indicate strong evidence for the null hypothesis, between 1/10 and 1/3 indicate moderate evidence for the null hypothesis, and between 1/3 and 3 indicate that the data are insensitive (based on Jeffreys’ Bayes factor cut‐offs [23, 26]).  *Moderate evidence for the null hypothesis; **strong evidence for the null hypothesis. | | | | | | |
