# Supplementary material for: Are smokers who are regularly exposed to e-cigarette use by others more or less motivated to stop or to make a quit attempt? A cross-sectional and longitudinal survey
Source: BMC Med. 2018 Nov 14;16:206. doi: 10.1186/s12916-018-1195-3 (PMC6234626; doi:10.1186/s12916-018-1195-3)
Supplement: Supplementary file 2 — Table S2. Unadjusted and adjusted associations between exposure to e-cigarette use in others and past quit attempts, high motivation to stop smoking and prospective quit attempts. (DOCX 21 kb) [file 12916_2018_1195_MOESM2_ESM.docx]

| **Table S2** Unadjusted and adjusted associations between exposure to e-cigarette use in others and past quit attempts, high motivation to stop smoking and prospective quit attempts | | | | | | | | | | |
| --- | --- | --- | --- | --- | --- | --- | --- | --- | --- | --- |
|  |  | **Past quit attempts**  **(*n*=12,787)** | |  | **High motivation to stop smoking (*n*=12,787)** | |  | **Prospective quit attempts  (*n*=1,580)** | |  |
|  |  | **RR_adj_ [95% CI]** | ***p*** |  | **RR_adj_ [95% CI]** | ***p*** |  | **RR_adj_ [95% CI]** | ***p*** |  |
| **Unadjusted models** | |  |  |  |  |  |  |  |  |  |
| Exposure to cigarette or  e-cigarette use by others | |  |  |  |  |  |  |  |  |  |
|  | Neither | 1.00 | - |  | 1.00 | - |  | 1.00 | - |  |
|  | Cigarettes only | 0.99 [0.90-1.08] | 0.764 |  | 0.90 [0.81-1.01] | 0.080 |  | 1.06 [0.84-1.34] | 0.635 |  |
|  | E-cigarettes only | 1.33 [1.07-1.63] | 0.009 |  | 1.45 [1.13-1.88] | 0.004 |  | 0.80 [0.42-1.49] | 0.476 |  |
|  | Both cigarettes and e-cigarettes | 1.18 [1.07-1.31] | 0.001 |  | 1.06 [0.93-1.20] | 0.414 |  | 1.19 [0.91-1.56] | 0.211 |  |
| **Adjusted models** | |  |  |  |  |  |  |  |  |  |
| Exposure to cigarette or  e-cigarette use by others | |  |  |  |  |  |  |  |  |  |
|  | Neither | 1.00 | - |  | 1.00 | - |  | 1.00 | - |  |
|  | Cigarettes only | 1.00 [0.91-1.10] | 0.974 |  | 0.92 [0.82-1.04] | 0.176 |  | 0.99 [0.78-1.26] | 0.948 |  |
|  | E-cigarettes only | 1.05 [0.85-1.30] | 0.654 |  | 1.19 [0.92-1.54] | 0.192 |  | 0.68 [0.36-1.28] | 0.231 |  |
|  | Both cigarettes and e-cigarettes | 0.96 [0.86-1.07] | 0.440 |  | 0.87 [0.76-1.00] | 0.051 |  | 0.99 [0.75-1.32] | 0.949 |  |
| Age | |  |  |  |  |  |  |  |  |  |
|  | 16-24 | 1.00 | - |  | 1.00 | - |  | 1.00 | - |  |
|  | 25-34 | 1.11 [0.99-1.24] | 0.090 |  | 1.24 [1.07-1.45] | 0.006 |  | 1.06 [0.73-1.54] | 0.777 |  |
|  | 35-44 | 1.05 [0.93-1.19] | 0.419 |  | 1.34 [1.14-1.58] | <0.001 |  | 0.91 [0.62-1.32] | 0.611 |  |
|  | 45-54 | 0.89 [0.79-1.01] | 0.070 |  | 1.17 [0.99-1.38] | 0.060 |  | 0.92 [0.65-1.30] | 0.628 |  |
|  | 55-64 | 0.83 [0.73-0.95] | 0.006 |  | 0.96 [0.81-1.15] | 0.666 |  | 0.67 [0.46-0.97] | 0.062 |  |
|  | 65+ | 0.71 [0.62-0.82] | <0.001 |  | 0.68 [0.56-0.84] | <0.001 |  | 0.65 [0.44-0.95] | 0.024 |  |
| Sex | |  |  |  |  |  |  |  |  |  |
|  | Men | 1.00 | - |  | 1.00 | - |  | 1.00 | - |  |
|  | Women | 1.10 [1.02-1.19] | 0.013 |  | 1.16 [1.05-1.28] | 0.003 |  | 1.02 [0.83-1.25] | 0.870 |  |
| Social grade | |  |  |  |  |  |  |  |  |  |
|  | ABC1 | 1.00 | - |  | 1.00 | - |  | 1.00 | - |  |
|  | C2DE | 0.93 [0.86-1.00] | 0.050 |  | 0.83 [0.75-0.91] | <0.001 |  | 0.87 [0.71-1.07] | 0.195 |  |
| Own e-cigarette use† | |  |  |  |  |  |  |  |  |  |
|  | No | 1.00 | - |  | 1.00 | - |  | 1.00 | - |  |
|  | Yes | 2.14 [1.97-2.33] | <0.001 |  | 1.95 [1.75-2.18] | <0.001 |  | 1.44 [1.13-1.82] | 0.003 |  |
| Survey year | | 1.00 [0.97-1.04] | 0.906 |  | 1.02 [0.98-1.07] | 0.395 |  | 0.95 [0.80-1.12] | 0.522 |  |
| RR = risk ratio; CI = confidence interval.  † Assessed with the question: ‘Are you using any of the following either to help you stop smoking, to help you cut down or for any other reason at all?’ | | | | | | | | | | |
